# Geometry Design Optimization of Functionally Graded Scaffolds for Bone Tissue Engineering: A Mechanobiological Approach

**DOI:** 10.1371/journal.pone.0146935

**Published:** 2016-01-15

**Authors:** Antonio Boccaccio, Antonio Emmanuele Uva, Michele Fiorentino, Giorgio Mori, Giuseppe Monno

**Affiliations:** 1 Dipartimento di Meccanica, Matematica e Management, Politecnico di Bari, 70126, Bari, Italy; 2 Dipartimento di Medicina Clinica e Sperimentale, Università di Foggia, 71122, Foggia, Italy; University of Akron, UNITED STATES

## Abstract

Functionally Graded Scaffolds (FGSs) are porous biomaterials where porosity changes in space with a specific gradient. In spite of their wide use in bone tissue engineering, possible models that relate the scaffold gradient to the mechanical and biological requirements for the regeneration of the bony tissue are currently missing. In this study we attempt to bridge the gap by developing a mechanobiology-based optimization algorithm aimed to determine the optimal graded porosity distribution in FGSs. The algorithm combines the parametric finite element model of a FGS, a computational mechano-regulation model and a numerical optimization routine. For assigned boundary and loading conditions, the algorithm builds iteratively different scaffold geometry configurations with different porosity distributions until the best microstructure geometry is reached, i.e. the geometry that allows the amount of bone formation to be maximized. We tested different porosity distribution laws, loading conditions and scaffold Young’s modulus values. For each combination of these variables, the explicit equation of the porosity distribution law–i.e the law that describes the pore dimensions in function of the spatial coordinates–was determined that allows the highest amounts of bone to be generated. The results show that the loading conditions affect significantly the optimal porosity distribution. For a pure compression loading, it was found that the pore dimensions are almost constant throughout the entire scaffold and using a FGS allows the formation of amounts of bone slightly larger than those obtainable with a homogeneous porosity scaffold. For a pure shear loading, instead, FGSs allow to significantly increase the bone formation compared to a homogeneous porosity scaffolds. Although experimental data is still necessary to properly relate the mechanical/biological environment to the scaffold microstructure, this model represents an important step towards optimizing geometry of functionally graded scaffolds based on mechanobiological criteria.

## Introduction

Functionally Graded Scaffolds (FGSs) for bone tissue engineering are porous biomaterials where the porosity changes with a specific gradient in space. The gradation of porosity enables FGSs to combine together the best mechanical properties of the denser material with those of the more porous one and the resulting material exhibits performances higher than those of the single constitutive materials. Low porosity regions offer high mechanical strength, high porosity regions promote, instead, cell adhesion and support cell growth, proliferation and differentiation [1–2].

Such scaffolds have been successfully utilized in the most variegated domains including the repair of long bone [1,3] and osteochondral [4–5] defects, the maxillofacial [6–7] and the spinal [8] surgery, the cranial reconstruction [9] and the drug delivery systems [1,10]. A large number of studies [11–13] are reported in the literature on the manufacturing processes that can be adopted to fabricate these biomaterials. Among the others, the strategy based on the integration of additive manufacturing or rapid prototyping techniques with computer-aided design models seems to be one of the most efficient [2,14]. The possibility of building any scaffold architecture with any type of porosity gradation and the experimental evidence that the geometry of porous scaffolds significantly influences the cellular response and the rate of bone tissue regeneration [15–17] led research community to find the possible models that relate the scaffold gradient to the mechanical and biological requirements for the regeneration of the bony tissue [2]. However, to date such models have not been developed yet.

In this article, we attempt to bridge the gap and propose a mechanobiology-driven optimization algorithm that, based on the boundary and loading conditions acting on the scaffold, identifies the best porosity distribution that allows the bone formation to be maximized. Other studies reported in the literature utilized optimization techniques to determine the best scaffold geometry [18–23] but none of them adopted mechanobiological criteria and determined the optimal porosity gradient in FGSs. In a previous study [24], the algorithm was utilized to determine the optimal pore dimension in regular structured open-porous scaffolds with homogeneous porosity. In the present study, the model was further developed to include a functionally graded porosity. In particular, three different variables have been investigated: the porosity distribution law, the loading conditions and the scaffold Young’s modulus; for each combination of the three variables, the algorithm determines the explicit equation of the porosity distribution law (i.e. the law that describes the pore dimensions in function of the spatial coordinate), that allows the largest volume of the scaffold to be occupied by bone.

## Materials and Methods

### Parametric model of an open-porous functionally graded scaffold

**Fig 1:**
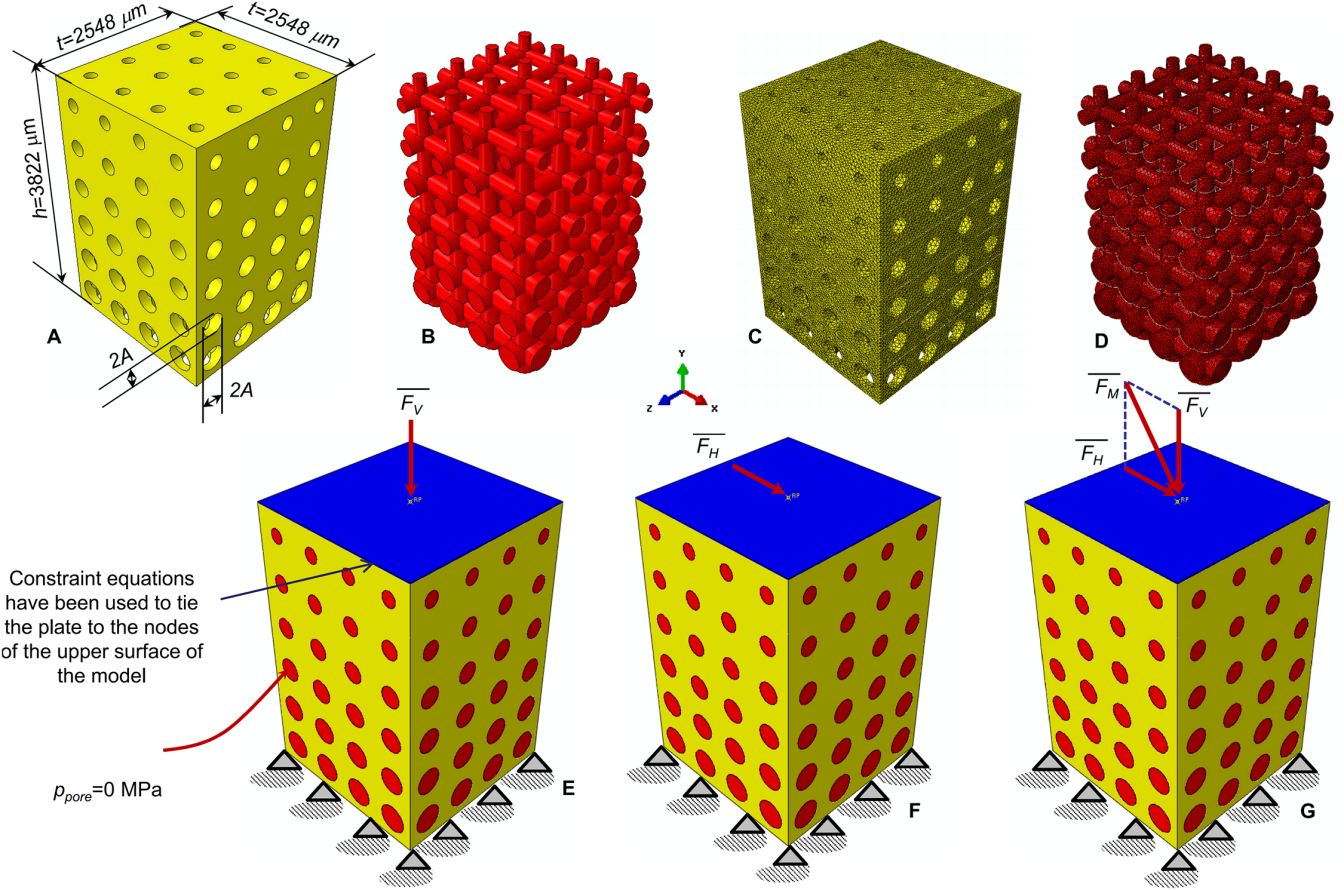
Parametric finite element model of the functionally graded scaffold utilized in the study. CAD model (A-B) and finite element mesh (C-D) of the scaffold (A, C) and granulation tissue (B, D). Circular pores with variable radius *A* have been modelled. The nodes of the bottom surface of the model were clamped (E-G) while those of the upper surface were tied to a rigid plate (represented in blue). Three different loading conditions were hypothesized: a compression force $\bar{F_{V}}$ (E); a shear force $\bar{F_{H}}$ (F); a mixed compression-shear force $\bar{F_{M}}$ (G). The pore pressure *p*_*pore*_ on the outer surfaces of the granulation tissue was set equal to zero to simulate the free exudation of fluid.

The parametric finite element model of an open-porous functionally graded scaffold was created in ABAQUS CAE^®^ Version 6.12 (Dassault Systèmes, France). The model has a prismatic geometry with a square *t* × *t* = 2548 μm × 2548 μm base and a *h* = 3822 μm height. The scaffold (represented in yellow, Fig 1A) includes circular pores with a parametric radius *A* that was assumed to change only along the *y* direction and remain constant along *x* and *z* direction (Fig 1A and 1B). According to Byrne et al. [25], the scaffold pores were hypothesized to be occupied by granulation tissue (represented in red, Fig 1B). The finite element mesh includes tetrahedral biphasic poro-elastic elements. 4-node linear coupled pore pressure elements (C3D4P) available in ABAQUS were utilized to model both, the scaffold (Fig 1C) and the granulation tissue (Fig 1D). The approximate element size was fixed equal to 40 μm.

Material properties implemented in the finite element model of the granulation tissue are the same as those utilized in previous studies [24, 26–27]. In detail, the Young’s modulus was set equal to 0.2 MPa; the permeability to 1×10^−14^ m^4^/N/s; the Poisson’s ratio to 0.167; the porosity to 0.8; the bulk modulus grain to 2300 MPa; the bulk modulus fluid to 2300 MPa. In order to evaluate the effect of the scaffold mechanical properties on the optimal porosity distribution, three different values of the Young’s modulus *E* were hypothesized: 500, 1000 and 1500 MPa which are the same as those utilized in a previous study [24].

The nodes of the bottom surface of the model were clamped (Fig 1E, 1F and 1G) while those of the upper surface were tied to a rigid plate (represented in blue, Fig 1E, 1F and 1G). For the outer nodes of the granulation tissue the pore pressure was fixed equal to 0 MPa which indicates that the liquid can freely exudate while applying the load. Three different loading conditions were hypothesized in the study: (a) a compression force ${\bar{F}}_{V}$ producing a vertical distributed load of *F*_*V*_ / (*t* × *t*) = 1 MPa (Fig 1E); (b) a shear force ${\bar{F}}_{H}$ producing an horizontal distributed load of *F*_*H*_ / (*t* × *t*) = 0.5 MPa (Fig 1F); (c) a mixed compression-shear force ${\bar{F}}_{M}$ given by the sum ${\bar{F}}_{M}={\bar{F}}_{V}+\bar{F_{H}}$ (Fig 1G). The choice of setting *F*_*H*_ = 0.5 × *F*_*V*_ was done because scaffolds are primarily designed to undergo to compression loading [25]. In all the hypothesized loading conditions, force was ramped over a time period of 1 s that is the possible time in which, a human body motion (such as to assume the erect position or to perform any motion of anatomical regions where a FGS can be implanted), can be completed. The same time interval was utilized in previous studies [24, 28–29].

### Porosity distribution laws

The dimension of the circular pores was controlled by the parametric radius *A* (Fig 1) that was hypothesized to change along the *y*-direction according to different porosity distribution laws. The coefficients of these distribution laws and hence their gradients were determined via the optimization algorithm described below. The porosity distribution laws considered in the study are the following: constant, linear, bi-linear and tri-linear.

**Fig 2:**
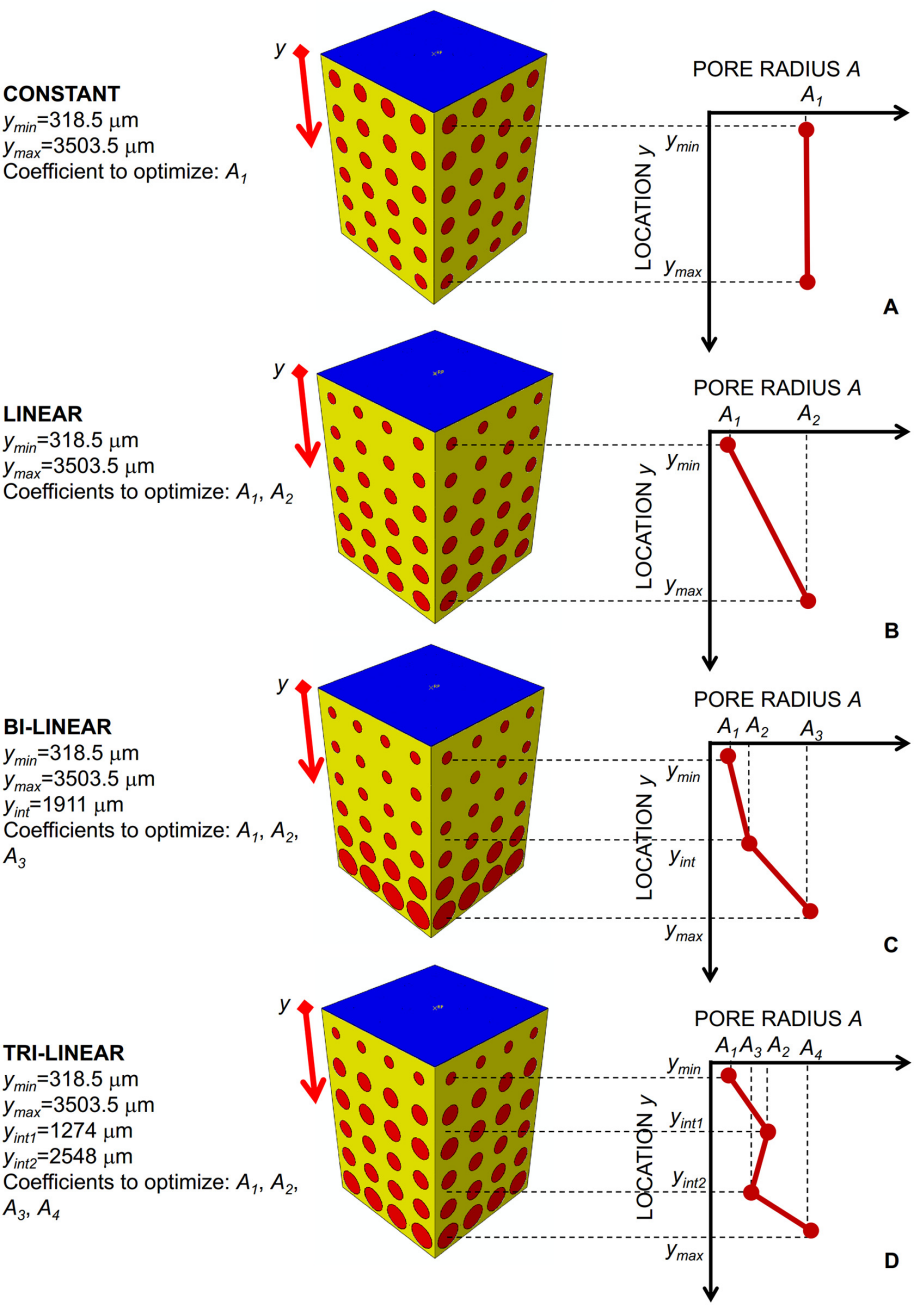
Porosity distribution laws analyzed in the study. (A) constant; (B) linear; (C) bi-linear; (D) tri-linear. The specific coefficients *A*_*i*_ (*i* = 1, 2, 3, 4) of these laws were determined via the optimization algorithm.

- Constant law. All the pores have the same dimensions (Fig 2A). In this case, the optimization algorithm determines just one coefficient, i.e. *A*_*1*_, that is the pore radius of all the scaffold pores.
- Linear law. The dimensions of pore change linearly with *y*. Two coefficients have to be determined by the optimization algorithm: *A*_*1*_ and *A*_*2*_ that are the pore radii at *y* = *y*_*min*_ and *y* = *y*_*max*_, respectively (Fig 2B).
- Bi-linear law. The pore radius *A* changes in the ranges [*y*_*min*_ *y*_*int*_] and [*y*_*int*_ *y*_*max*_] with two different linear laws that assume the same value for *y* = *y*_*int*_. The coefficients to optimize are three: *A*_*1*_, *A*_*2*_ and *A*_*3*_ (Fig 2C).
- Tri-linear law. The dimensions of the pore change in the intervals [*y*_*min*_ *y*_*int1*_], [*y*_*int1*_ *y*_*int2*_], [*y*_*int2*_ *y*_*max*_] with three different linear laws. The laws defined in the first and second and those defined in the second and third interval assume the same value for *y* = *y*_*int1*_ and for *y* = *y*_*int2*_, respectively. In this case, the optimization algorithm determines four coefficients: *A*_*1*_, *A*_*2*_, *A*_*3*_ and *A*_*4*_ (Fig 2D).

The specific values of *y*_*max*_, *y*_*min*_, *y*_*int*_, *y*_*int1*_ and *y*_*int2*_ are reported in Fig 2. It is worthy to note that, once the coefficients *A*_*i*_ (*i* = 1, 2, 3, 4) have been determined, the explicit equation of the best porosity distribution, i.e. the equation that describes how the pore radius *A* changes with *y*, can be obtained by simply implementing the obtained coefficients in the relationships reported in Table 1.

**Table 1:** Porosity distribution laws implemented in the study.

*Porosity distribution*	*Coefficients to optimize*	*Equation*	*Gradient*
Constant law	*A*_1_	*A* = *A*_1_	0
Linear law	*A*_1,_*A*_2_	*A* = *A*_2_+*m*(*y*−*y*_max_)	$m=\frac{A_{2}-A_{1}}{y_{\text{max}}-y_{\text{min}}}$
Bi-linear law	*A*_1,_*A*_2,_*A*_3_	*for y*∈[*y*_min_ *y*_int_]⇒*A* = *A*_2_+*m*_1_(*y*−*y*_int_) *for y*∈[*y*_min_ *y*_int_]⇒*A* = *A*_3_+*m*_2_(*y*−*y*_max_)	$\begin{array}{ll} m_{1} & =\frac{A_{2}-A_{1}}{y_{\text{int}}-y_{\text{min}}}\\ m_{2} & =\frac{A_{3}-A_{2}}{y_{\text{max}}-y_{\text{int}}} \end{array}$
Tri-linear law	*A*_1,_*A*_2,_*A*_3,_*A*_4_	*for y*∈[*y*_min_ *y*_int1_]⇒*A* = *A*_2_+*m*_1_×(*y*−*y*_int1_) *for y*∈[*y*_int1_ *y*_int2_]⇒*A* = *A*_3_+*m*_2_×(*y*−*y*_int2_) *for y*∈[*y*_int2_ *y*_max_]⇒*A* = *A*_4_+*m*_3_×(*y*−*y*_max_)	$\begin{array}{ll} m_{1} & =\frac{A_{2}-A_{1}}{y_{\text{int}1}-y_{\text{min}}}\\ m_{2} & =\frac{A_{3}-A_{2}}{y_{\text{int}2}-y_{\text{int}1}}\\ m_{3} & =\frac{A_{4}-A_{3}}{y_{\text{max}}-y_{\text{int}2}} \end{array}$

### Computational mechano-regulation model

Once the mesenchymal stem cells invade the scaffold and spread through its pores, the bone regeneration process starts. After dispersal, cells will differentiate. The biophysical stimulus *S* that regulates the differentiation process was hypothesized to be a function of the octahedral shear strain *ɣ* and interstitial fluid flow *ʋ* in the extracellular environment of the cells. In detail, let *ɛ*_*I*_, *ɛ*_*II*_, and *ɛ*_*III*_ be the principal strains, the octahedral shear strain *ɣ* can be defined as:

$$\gamma =\frac{1}{2}\sqrt{{\left(\varepsilon_{I}-\varepsilon_{II}\right)}^{2}+{\left(\varepsilon_{II}-\varepsilon_{III}\right)}^{2}+{\left(\varepsilon_{III}-\varepsilon_{I}\right)}^{2}}$$ (1)

Calling *a* and *b* two empirical constants defined as in Huiskes et al. [30], and given by *a* = 3.75% and *b* = 3 μms^-1^, the biophysical stimulus *S* can be expressed, according to Prendergast et al. [31], as:

$$S=\frac{\gamma }{a}+\frac{v}{b}$$ (2)

Mesenchymal stem cells differentiate into different cell phenotypes according to the following inequalities:

$$\left\{ \begin{array}{l} if\;S>c\Rightarrow fibrogenesis\Rightarrow fibroblasts\Rightarrow fibrous_{{}_{}}{}_{}tissue_{}{}_{}formation\\ if\;1<S<c\Rightarrow condrogenesis\Rightarrow chondrocytes\;\Rightarrow \;cartilagineous_{}{}_{}tissue_{}{}_{}formation\\ if\;n_{mature}<S<1\Rightarrow osteogenesis\Rightarrow osteoblasts\;\Rightarrow \;immature_{}{}_{}bone_{}{}_{}tissue_{}{}_{}formation\\ if\;n_{resorb}<S<n_{mature}\Rightarrow osteogenesis\Rightarrow osteoblasts\;\Rightarrow \;mature_{}{}_{}bone_{}{}_{}tissue_{}{}_{}formation\\ if\;0<S<n_{resorb}\Rightarrow osteoclasts\;\Rightarrow \;bone_{}{}_{}resorbtion \end{array}\right.$$ (3)

where *n*_*resorb*_ = 0.01, *n*_*mature*_ = 0.53 and *c* = 3 represent boundaries of the mechano-regulation diagram the values of which are the same as those utilized in other studies [28, 32–33].

### Optimization algorithm

**Fig 3:**
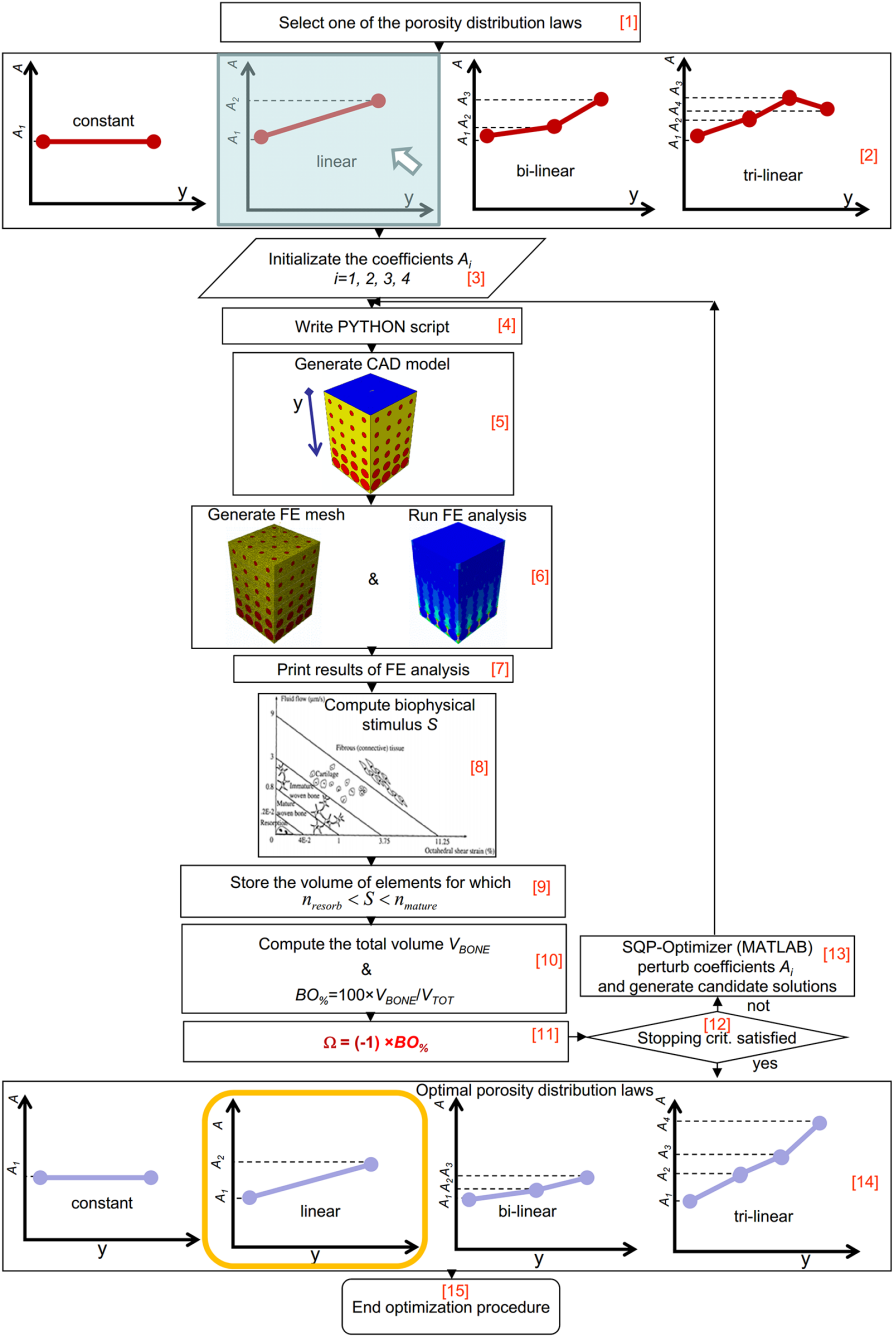
Schematic of the algorithm implemented in Matlab environment to optimize the porosity distribution law in functionally graded scaffolds.

The FGS parametric finite element model, the computational mechano-regulation model above described and a numerical optimization routine were combined together in an algorithm written in Matlab^®^ (v. R2011b) (Fig 3) that aims to determine, for each of the hypothesized scaffold Young’s moduli, loading conditions and porosity distribution laws, the equations of the best porosity distribution that allows the bone formation to be maximized. Considering that 3 scaffold Young’s modulus values (i.e. 500, 1000 and 1500 MPa), 3 loading conditions (i.e. ${\bar{F}}_{V}$,${\bar{F}}_{H}$, and ${\bar{F}}_{M}$) and 4 porosity distribution laws (i.e. constant, linear, bi-linear and tri-linear) have been hypothesized, it follows that a total of 3 × 3 × 4 = 36 optimization analyses have been performed in the study.

As a first step, the algorithm requires to select (Block [1]) one of the porosity distribution laws (Block [2]). The initialization of coefficients *A*_*i*_ follows (Block [3]), the user can assign to *A*_*i*_ initial values that fall within the interval [*A*_*lower*_ *A*_*upper*_], where *A*_*lower*_ = 5 μm and *A*_*upper*_ = 300 μm have been taken the same as those utilized in a previous study [24]. The algorithm implements the specified initial values of *A*_*i*_ into a PYTHON script (Block [4]) that is given in input to ABAQUS. The PYTHON script, based on the values *A*_*i*_, defines in function of the coordinate location *y* the dimension of each pore. The module ABAQUS CAE builds the CAD model of the functionally graded scaffold (Block [5]) with the computed pore dimensions, and after applying the boundary and (one of) the (three) loading conditions above described, generates the finite element mesh (Block [6]). The finite element analysis follows that accounts for geometrical and material nonlinearities (Block [6]). For each element occupying the scaffold pores, i.e. the elements represented in red in Fig 1D, ABAQUS prints (Block [7]) the values of the principal strains *ɛ*_*I*_, *ɛ*_*II*_ and *ɛ*_*III*_ and of the interstitial fluid flow *ʋ* that the algorithm utilizes to compute, through the eqs (1) and (2), the magnitude of the biophysical stimulus *S* (Block [8]). Then, the relationships eq (3) are implemented and for those elements for which the inequality

$$n_{resorb}<S<n_{mature}$$ (4)

is satisfied, i.e. for those elements where the formation of mature bone is predicted to take place, the volume *V*_*i_bone*_ is stored (Block [9]). If *n*_*b*_ is the number of elements where inequality eq (4) is satisfied, the algorithm calculates the total volume of these elements as:

$$V_{BONE}=\overset{nb}{\underset{i=1}{\sum }}V_{i\_bone}$$ (5)

If *V*_*TOT*_ is the total volume of the scaffold model *V*_*TOT*_ = *t* × *t* × *h* = 2548 μm × 2548 μm × 3822 μm = 24.814 mm^3^, the algorithm determines the percentage of scaffold volume *BO*_*%*_ that is occupied by bone as (Block [10]):

$$BO_{\%}=\frac{V_{BONE}}{V_{TOT}}\times 100$$ (6)

and calculates the value of the objective function Ω as (Block [11]):

$$\Omega =(-1)\times BO_{\%}$$ (7)

At this point, the algorithm formulates an optimization problem that includes the coefficients *A*_*i*_ as design variables and that aims to minimize the value of the objective function Ω or, equivalently, to maximize the percentage *BO*_*%*_ of volume occupied by bone. It can be claimed, in fact, that the greater the efficiency of the scaffold, the larger the amount of bone produced by the scaffold itself. In an ideal scaffold, 100% of its volume is occupied by bone. The inverse problem described with the eqs (6) and (7) was solved with the Sequential Quadratic Programming (SQP) method available in Matlab, an iterative method for nonlinear optimization. The number of iterations performed by the method can be controlled by means of specific stopping criteria that can be selected by the user and that include a number of tolerances. As one of these stopping criteria is meet, the optimization process ends and after implementing the optimal coefficients *A*_*i*_ in the relationships of Table 1, the optimal porosity distributions are traced in function of *y* (Blocks [14] and [15]). If no stopping criteria are satisfied, the optimization algorithm assigns new values to *A*_*i*_ thus generating new candidate solutions (Block [13]). The optimization process terminates when one of the selected stopping criteria is satisfied (Block [12]).

The biophysical stimulus *S* on which the objective function Ω depends, was computed based on the hypothesis that the dispersal of mesenchymal stem cells has already taken place and that the only granulation tissue, with the mechanical properties above described, occupies the scaffold pores.

All the computations were performed on a HP Z620- Intel^®^ Xeon^®^ Processor E5-2620—16Gb RAM. The most expensive optimization analyses were those implementing the tri-linear law that took around 300 hours of computations.

## Results

**Fig 4:**
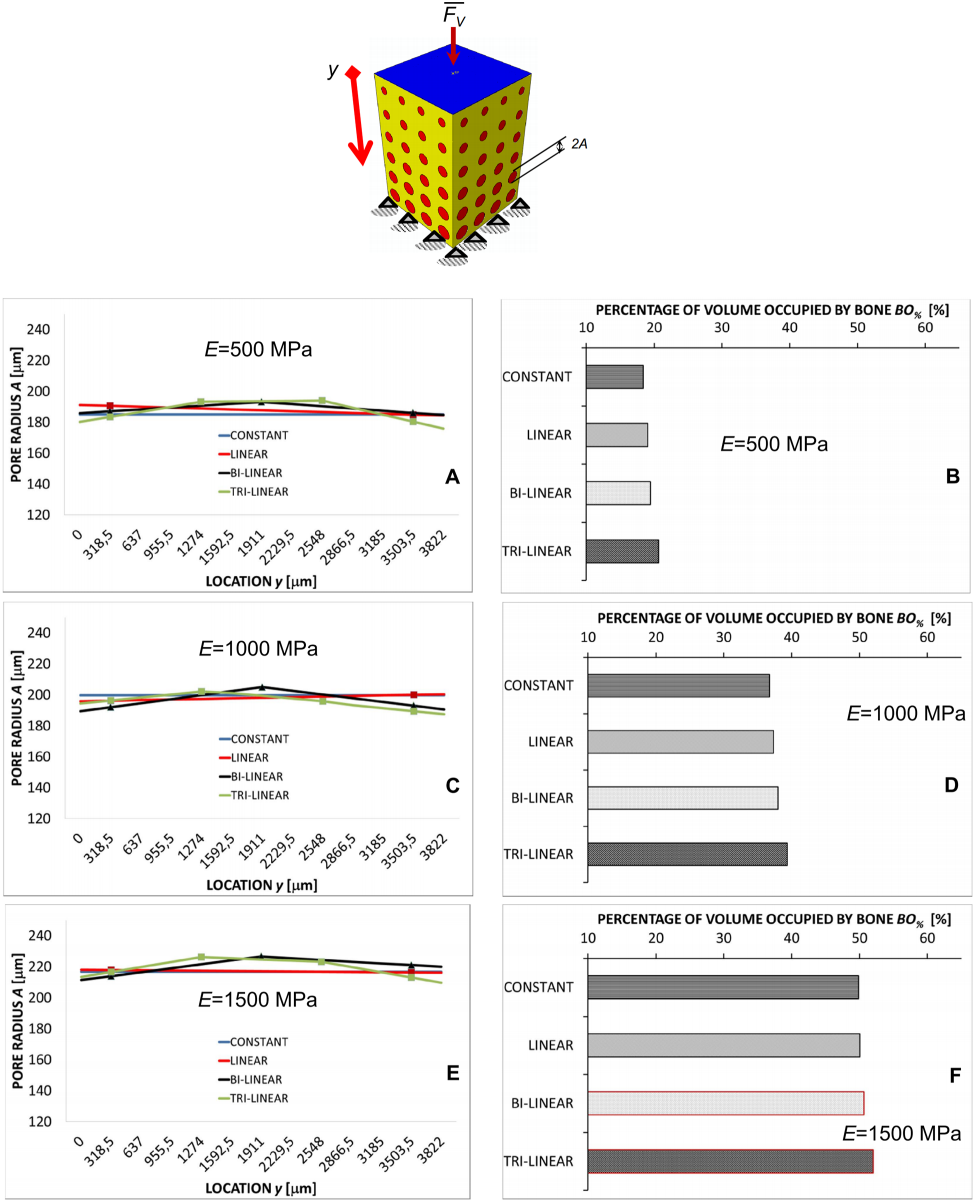
Computed values of *A* and *BO*_*%*_ in the case of compression loading. Pore radius *A* (A, C, E) (vs. location *y*) and percentages of the scaffold volume occupied by bone *BO*_*%*_ (B, D, F) predicted by the optimization algorithm in the case of compression loading *F*_*V*_ for different scaffold Young’s moduli and after implementing different porosity distribution laws. The schematic figure shown on the top indicates the loading condition to which the diagrams refer. All the values of *BO*_*%*_ reported in the diagrams refer to the optimal configuration, i.e. the configuration for which Ω reaches its minimum value.

In the case of the compression loading *F*_*V*_ the predicted pore dimension experiences small changes (Fig 4A, 4C and 4E) along the *y*-axis and is almost constant. Independently from the scaffold Young’s modulus *E*, *A* does not change by more than 15 μm. The general trend (with the exception for the porosity distribution obtained implementing the constant law) that can be observed is that the pore radius in the vicinity of the clamps (i.e. for high values of *y*) and of the load (i.e. for small values of *y*) slightly decreases. For increasing values of *E*, the pore radius, on average, increases. For instance, in the case of *E* = 500 MPa, the average pore radius is about 190 μm, for *E* = 1500 MPa, instead, becomes about 220 μm. The percentage of volume occupied by bone *BO*_*%*_ increases as we move from the constant to the tri-linear porosity distribution (Fig 4B, 4D and 4F). Furthermore, increasing values of *BO*_*%*_ were predicted for increasing values of the scaffold Young’s modulus (Fig 4B, 4D and 4F).

**Fig 5:**
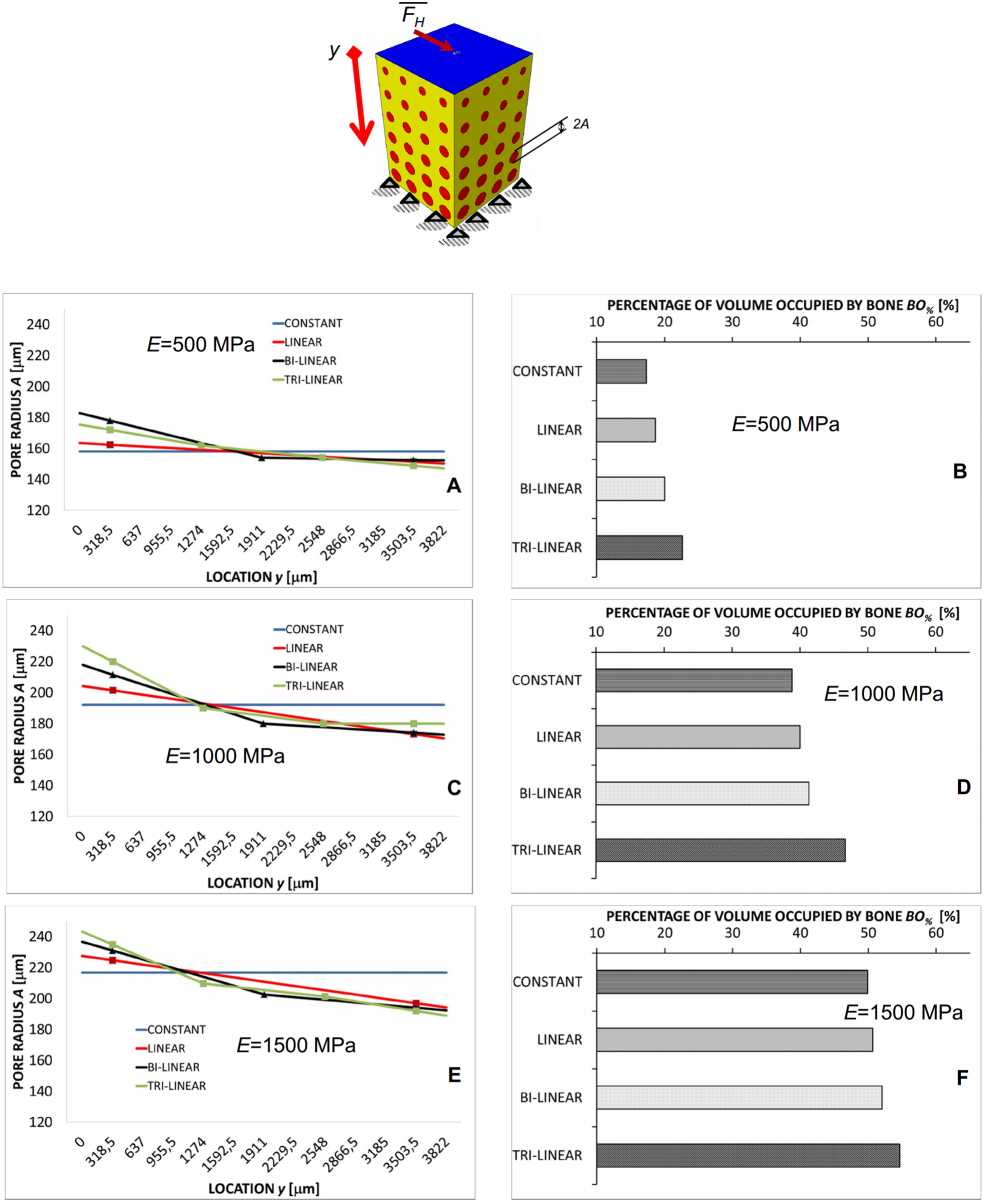
Computed values of *A* and *BO*_*%*_ in the case of shear loading. Pore radius *A* (A, C, E) (vs. location *y*) and percentages of the scaffold volume occupied by bone *BO*_*%*_ (B, D, F) predicted by the optimization algorithm in the case of shear loading *F*_*H*_ for different scaffold Young’s moduli and after implementing different porosity distribution laws. The schematic figure shown on the top indicates the loading condition to which the diagrams refer. All the values of *BO*_*%*_ reported in the diagrams refer to the optimal configuration, i.e. the configuration for which Ω reaches its minimum value.

More interesting appears the porosity distribution predicted by the algorithm in the case of the shear load *F*_*H*_ (Fig 5) where important changes of the pore dimensions are predicted along the *y*-axis (Fig 5A, 5C and 5E). The highest values of *A* are predicted in the vicinity of the load (i.e. for small values of *y*) while the pore dimensions tend to decrease as we move towards the clamped region. Also in this case *BO*_*%*_ increases as we move from the constant to the tri-linear porosity distribution, however, the change of *BO*_*%*_ is more significant than in the case of compression load. For increasing levels of *E*, the average value of *BO*_*%*_ increases too (Fig 5B, 5D and 5F).

**Fig 6:**
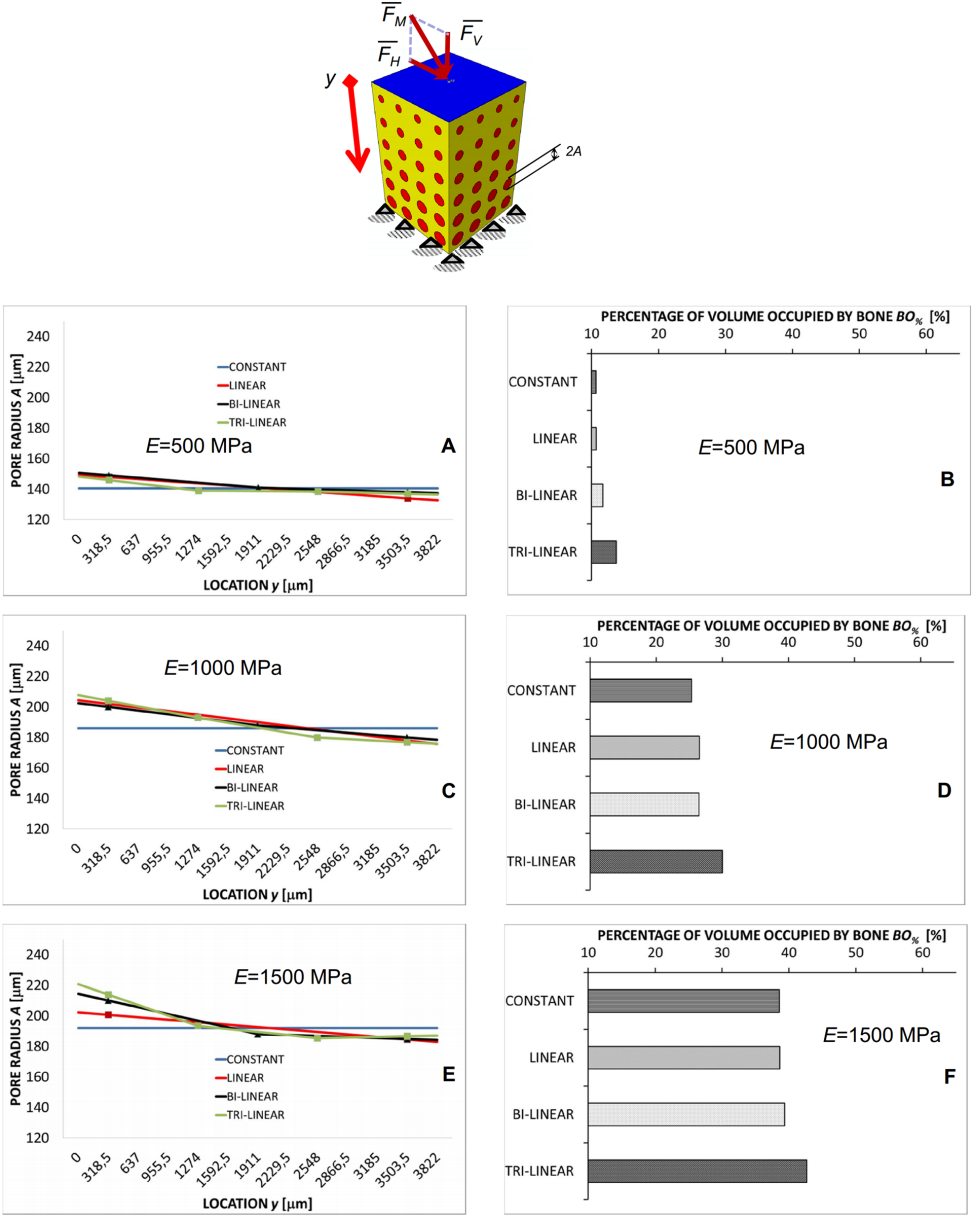
Computed values of *A* and *BO*_*%*_ in the case of mixed load. Pore radius *A* (a, c, e) (vs. location *y*) and percentages of the scaffold volume occupied by bone *BO*_*%*_ (b, d, f) predicted by the optimization algorithm in the case of mixed load *F*_*M*_ for different scaffold Young’s modulus values and after implementing different porosity distribution laws. The schematic figure shown on the top indicates the loading condition to which the diagrams refer. All the values of *BO*_*%*_ reported in the diagrams refer to the optimal configuration, i.e. the configuration for which Ω reaches its minimum value.

In the case of mixed load *F*_*M*_, the pore radius *A* experiences changes that are less important than those predicted in the case of shear load *F*_*H*_ but that are certainly larger than those computed in the case of compression load *F*_*V*_ (Fig 6A, 6C and 6E). As in the previous case, the pore dimension decreases for increasing values of *y*. *BO*_*%*_ increases as we move from the constant to the tri-linear law and its average value increases for increasing values of the scaffold Young’s modulus *E* (Fig 6B, 6D and 6F). For a fixed value of *E* and porosity distribution law, the values of *BO*_*%*_ predicted in the case of mixed load *F*_*M*_ are smaller than those predicted for the other hypothesized loading conditions (Figs 4B, 4D, 4F, 5B, 5D, 5F, 6B, 6D and 6F).

**Fig 7:**
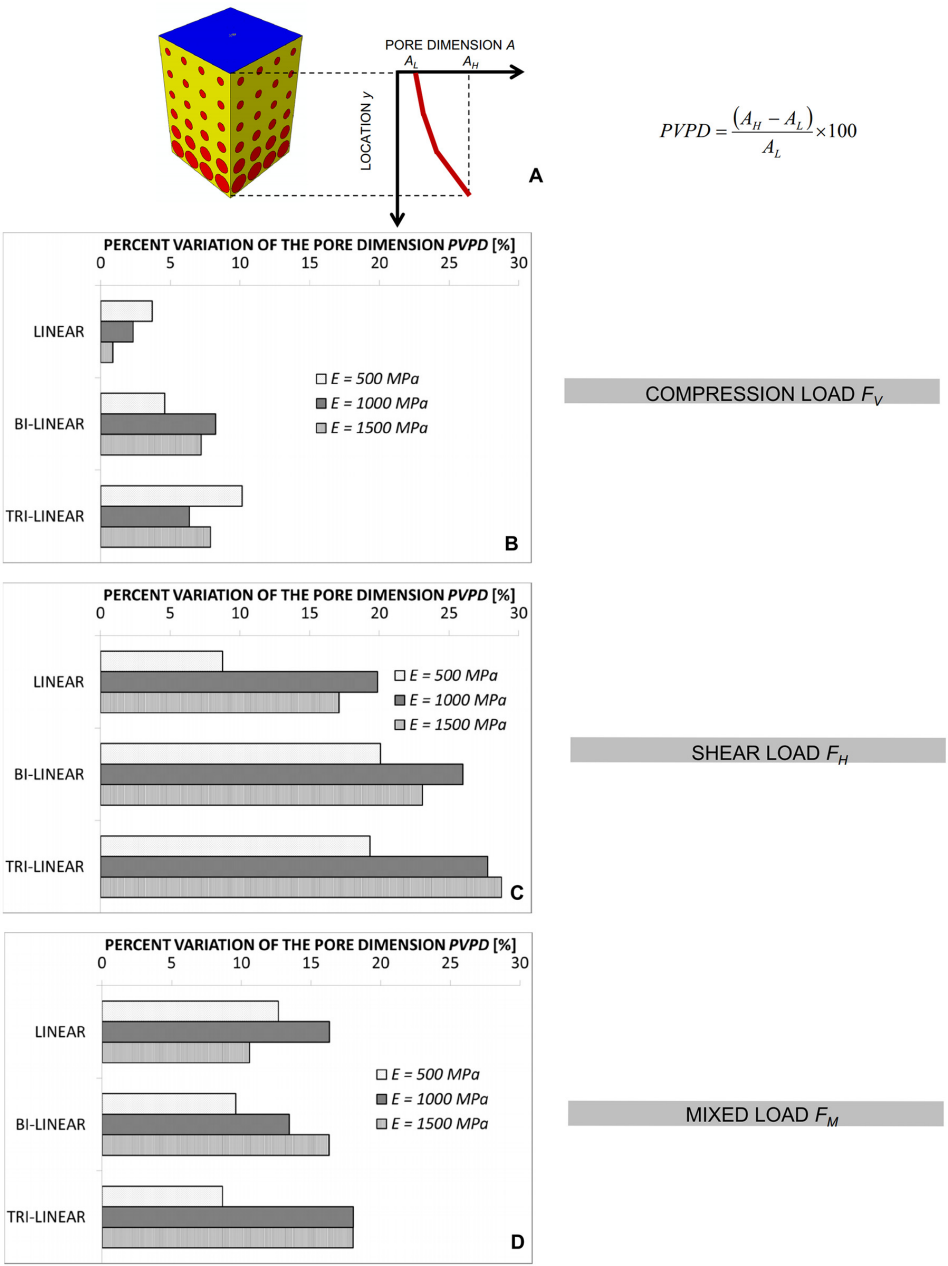
Computed values of *PVPD* for different loading conditions. Percent Variation of the Pore Dimension (*PVPD*) for the compression *F*_*V*_ (B), the shear *F*_*H*_ (C) and the mixed *F*_*M*_ (D) load and for all the hypothesized scaffold Young’s modulus values. (A) reference schematic utilized to calculate the parameter *PVPD*. Note: *A*_*H*_ and *A*_*L*_ are the highest and lowest value of *A* that can be located in correspondence of any value of *y* and not necessarily, as reported in the figure, of the furthest values *y* = 0 μm and *y* = *h* = 3822 μm.

In order to quantify (i) the change of the pore dimensions with *y* and (ii) the “usefulness” of utilizing a functionally graded scaffold instead of a scaffold with a homogenous porosity distribution we introduced two parameters. The first one, denoted as *PVPD*, represents the Percent Variation of the Pore Dimension and is defined as:

$$PVPD=\frac{\left(A_{H}-A_{L}\right)}{A_{L}}\times 100$$ (8)

where *A*_*H*_ and *A*_*L*_ are the highest and the lowest value of *A* along the *y*-axis, respectively (Fig 7A). In general, the higher the *PVPD*, the larger are the changes of the pore dimension *A*. The highest values of *PVPD* have been found in the case of the shear loading *F*_*H*_ (Fig 7) where changes of *A* also by more than 25–30% were predicted (Fig 7C). Slightly lower are the values of *PVPD* found in the case of the mixed load *F*_*M*_ (Fig 7D) and yet less significant those computed in the case of the compression load *F*_*V*_ (Fig 7B). Averagely, it appears that *PVPD* does not depend neither on the scaffold Young’s modulus *E*, nor on the porosity distribution law but does depend on the loading conditions. For the constant law, regardless of the type of load considered, the value of *PVPD* is zero and is not shown in Fig 7.

In general, it appears that as we move from the constant to the linear, bi-linear and, finally, tri-linear porosity distribution law the percentage of volume occupied by bone *BO*_*%*_ increases (Figs 4B, 4D and 4F, 5B, 5D and 5F, 6B, 6D and 6F). In particular, the highest values of *BO*_*%*_ have been found for the tri-linear law while the lowest ones for the constant law. Therefore, it makes sense to introduce the second parameter, denoted as *iBO*_*%*_ and defined as the increment of *BO*_*%*_ when we move from the constant to the tri-linear law. If *BO*_*%_tri-linear*_ is the percentage of volume occupied by bone predicted for the tri-linear porosity distribution and *BO*_*%_constant*_ the percentage predicted with the constant one, *iBO*_*%*_ can be expressed as:

$$iBO_{\%}=BO_{\%\_tri-linear}-BO_{\%\_constant}$$ (9)

**Fig 8:**
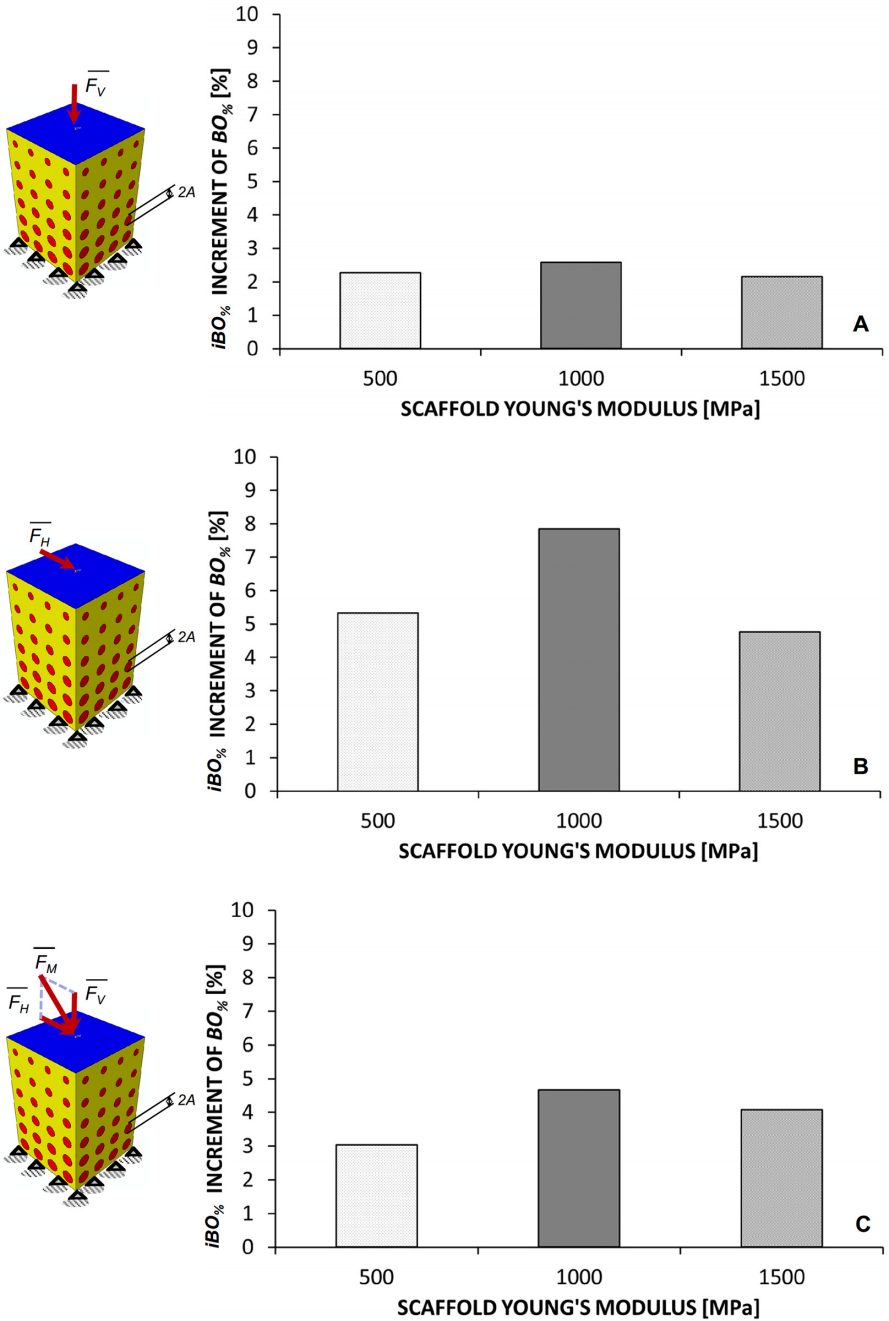
Computed values of *iBO*_*%*_ for compression (A), shear (B) and mixed (C) load.

As is clear, the higher the values of *iBO*_*%*_, the more “useful” is the utilization of a functionally graded scaffold instead of a homogeneous porosity scaffold. In the limit case where *iBO*_*%*_ = 0%, the use of a FGS does not make sense and a homogeneous porosity scaffold has the same potentialities of generating bone as the FG one. On average, the highest values of *iBO*_*%*_ were computed in the case of shear loading *F*_*H*_ followed by the mixed load *F*_*M*_ and the compression load *F*_*V*_, respectively (Fig 8). In particular, among the hypothesized scaffold Young’s moduli, the highest values of *iBO*_*%*_ were predicted for *E* = 1000 MPa.

**Fig 9:**
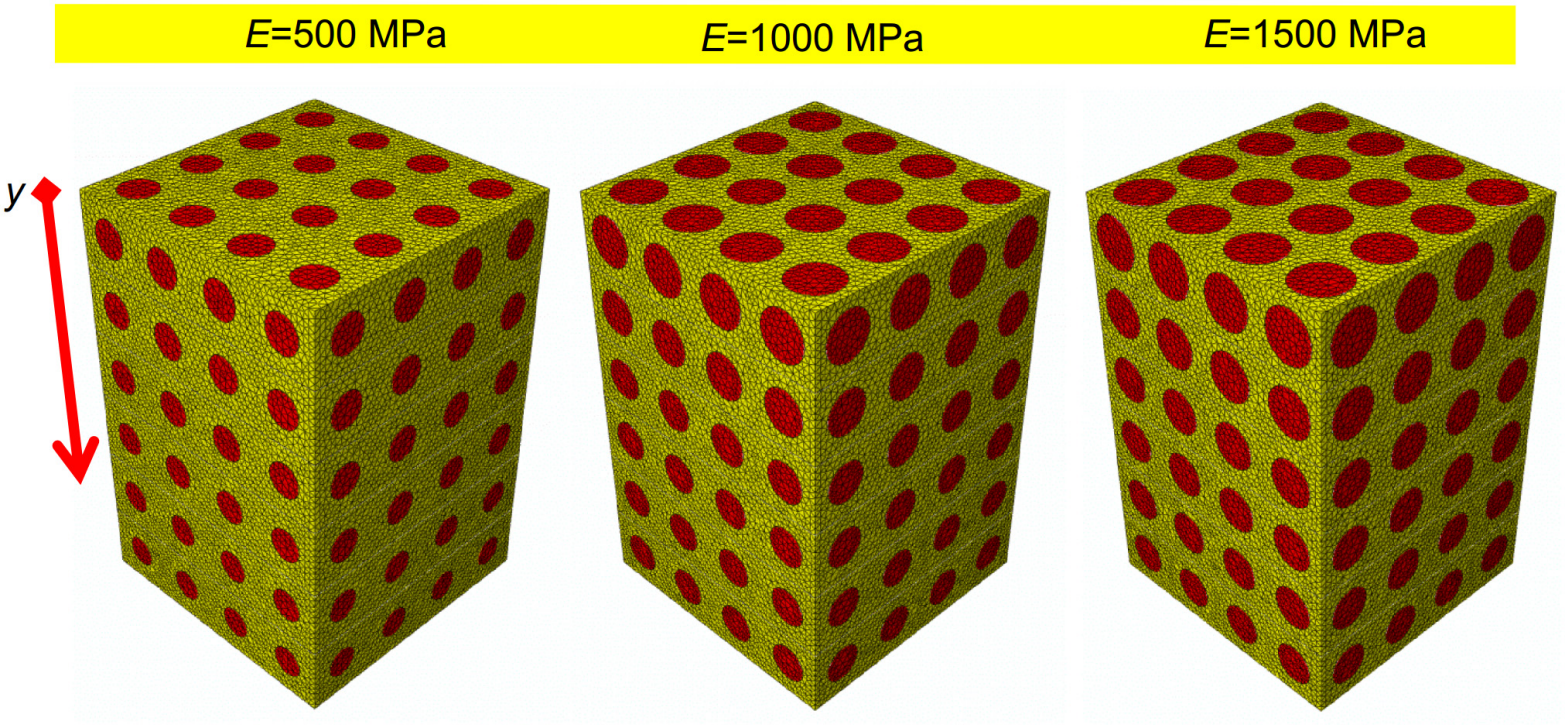
3D view of the best geometrical configurations (tri-linear porosity distribution) predicted by the optimization algorithm for the shear loading condition.

A three-dimensional view of the optimal scaffold geometry predicted for the tri-linear porosity distribution (that is the law with which the highest values of *BO*_*%*_ have been obtained) and the shear loading *F*_*H*_ is shown in Fig 9. As it can be seen, the pore dimensions change significantly along the *y*-axis and, on average, increase for increasing values of *E*.

## Discussion

This article presented an optimization algorithm based on mechanobiological criteria and aimed to determine the best porosity distribution in functionally graded scaffolds for bone tissue engineering.

Four *porosity distribution laws*, three *loading conditions* and three *scaffold Young’s moduli* were hypothesized. For each combination of these three variables, the optimal microstructure geometry was determined. It was shown that all these variables have a critical effect on the amounts of bone predicted to form within the scaffold pores.

Regarding the *porosity distribution law*, it was found that designing FGSs with a tri-linear law allows the largest amounts of bone to be generated (Figs 4–6) compared to bi-linear, linear and constant laws. In general, the use of porosity distribution laws with increasing complexity level (i.e. with increasing number of coefficients *A*_*i*_) leads the scaffold geometry to be better tailored to the specific boundary and loading conditions acting on the construct thus allowing the bone formation to be maximized. Increasing the complexity level of a porosity distribution means, in other words, to include a larger number of design variables and hence, to increase the probability that the optimizer will find a geometry that allows larger amounts of bone to be generated.

More critical appears the effect of the *loading conditions*. For a pure compression loading, the changes of the pore dimension *A* are marginal (Figs 4, 7 and 9) and using a FGS allows the formation of amounts of bone slightly larger than those obtainable with a homogeneous porosity scaffold (Fig 8). For a pure shear loading, instead, FGSs allow to significantly increase the bone formation compared to a homogeneous porosity scaffolds (Figs 5B, 5D, 5F and 8) and the pore dimensions change (vs. *y*) also by more than 20–25% (Figs 7 and 9). This behavior can be justified with the following argument. In the case of pure shear loading, strains increase as we move from the loaded towards the clamped region and hence, the stimulus *S*, that is a function of the strain, changes in the same manner. In order to maximize the number of elements for which inequality eq (4) is satisfied, the optimization solver tends to reduce the dimension of the pores subjected to higher strain and increase that of the pores subjected to lower strain. In the case of pure compression, instead, (from the macroscopic point of view) the scaffold model is subjected to an uniaxial stress state (with the only exception of the regions close to the loaded and the clamped surfaces where the stress state becomes tri-axial) and then to a more or less uniform distribution of the stimulus *S*, which explains the approximately uniform dimensions of the pores. The mixed load *F*_*M*_ leads to an intermediate situation between the pure compression and the pure shear. Changes of *A* as well increments of *BO*_*%*_ are more important than those predicted in the case of compression force *F*_*V*_ but less relevant than those computed with the shear load *F*_*H*_ (Figs 6–8).

Finally, regarding the *scaffold Young’s modulus* it appears that the average pore dimension *A* increases for increasing values of *E* (Fig 9). This can be justified with the argument that as the Young’s modulus increases, the global scaffold stiffness increase too and the optimizer tends to increase the dimensions of the pores to include larger amounts of bone.

To determine the optimal porosity distribution in FGSs some assumptions were made. First of all, the temporal variable was neglected. It was assumed that the scaffold pores are occupied only by granulation tissue, the processes of diffusion of the mesenchymal stem cells and of tissue differentiation were not simulated and the optimization of the porosity distribution was carried out based on the values of the biophysical stimulus registered at the initial time instant. Furthermore, the algorithm does not include scaffold resorption potential [25].

Including the time variable would certainly allow to carry out more accurate predictions on the best porosity distribution but would lead to a dramatic increase of the computational time thus making the algorithm practically not implementable in a “clinical” context. Other aspects such as angiogenesis [34–36] and growth factors [37] involved in the process of bone regeneration were not modelled. This model neglects the effect of loads during the initial development of a tissue on a scaffold, i.e. during the phase in which cell attach to the scaffold surface. The scaffold surface is a 2D environment while the model utilized in this study is based on volumetric strains. A model to predict the effect of mechanical signals on cells seeded on the surface of a scaffold has been reported [38]. Another limitation of the model is that a deterministic approach was adopted to determine the biophysical stimulus *S*, —on the definition of which the optimal porosity distribution law is calculated,—which neglects any possible genetic variability in animal populations. A more general and complete approach would be the probabilistic one and would take into account this variability.

**Fig 10:**
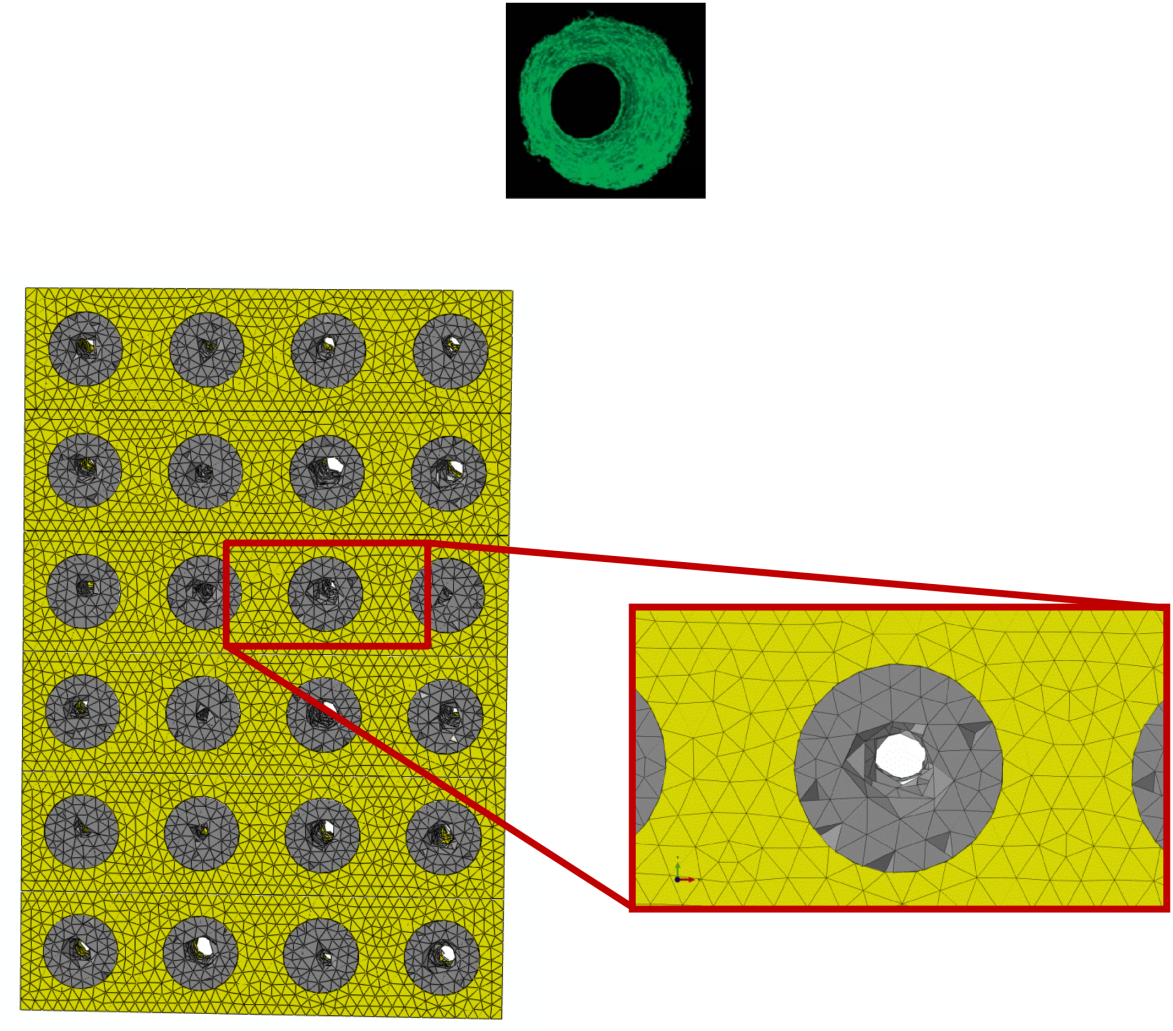
Patterns of bone predicted in the case of: (i) compression loading; (ii) scaffold Young’s modulus *E* = 1000 MPa; (iii) porosity distribution law: constant. Elements in gray are representative of the regions within the scaffold pores where the algorithm predicts bone formation. Interestingly, the predicted bony tissue patterns appear consistent with those of new tissue formed in three-dimensional matrix channels observed in an in vitro study [39]. Bone formation starts from the pore walls and propagates towards the pore center.

However, despite these limitations, the predictions of the model are consistent with the results of experimental studies. For instance, the patterns of bony tissue predicted in the case of a pure compression load, constant porosity distribution, *E* = 1000 MPa, are consistent with those of new tissue generated in circular matrix channels observed in histological analyses [39]. In vitro, it was found that, bone forms from the channel walls and tends to growth towards the center of the pore. This same behavior was observed in the numerical model (Fig 10). The grey elements shown in Fig 10 represent the volumes of the model where the mechano-regulation model predicts the formation of bone. Furthermore, as demonstrated in previous studies a minimum pore size of about 100 μm is required to guarantee a successful bone regeneration process in scaffolds [40]. The pore dimensions predicted by the model are all above this threshold value and well fall within the range of the typical dimensions of the pores of scaffolds for bone tissue engineering [41]. Other studies report that the rate of bone regeneration in scaffold is a function of the scaffold mechanical properties [42]. This is also consistent with the predictions of the present model where the amounts of bone *BO*_*%*_ change for changing values of the scaffold Young’s modulus (Figs 4–6 and 9).

## Conclusions

A mechanobiology-driven optimization algorithm was presented to determine the optimal porosity distribution in functionally graded scaffolds. The results presented in this paper show that the loading conditions are pivotal in determining optimal porosity distribution. For a pure compression loading, it was predicted that the changes of the pore dimension are marginal and using a FGS allows the formation of amounts of bone slightly larger than those obtainable with a homogeneous porosity scaffold. For a pure shear loading, instead, FGSs allow to significantly increase the bone formation compared to a homogeneous porosity scaffold. Increasing pore dimensions are predicted for increasing values of the scaffold Young’s modulus. Increasing the number of coefficients that define a porosity distribution law allows to design more performing scaffolds capable of generating larger amounts of bone.

The model predictions appear reasonably consistent with what is observed in vitro. Although experimental data is still necessary to properly relate the mechanical/biological environment to the scaffold microstructure geometry, this model represents an important step towards optimizing geometry of functionally graded scaffolds and/or stimulation regimes based on mechanobiological criteria.

## Abbreviations

t: length of the side of the basis of the scaffold prismatic model
h: height of the scaffold prismatic model
V_TOT_: total volume of the scaffold model
$\bar{F_{V}}$: compression force acting on the scaffold and producing a vertical distributed load
$\bar{F_{H}}$: shear force acting on the scaffold and producing a horizontal distributed load
$\bar{F_{M}}$: mixed compression-shear force acting on the scaffold
p_pore_: pore pressure acting on the outer surfaces of the granulation tissue
E: scaffold Young’s modulus
A: pore radius
*A*_*i*_ (*i* = 1, 2, 3, 4): pore radius at specific *y* locations
*y*_*max*_, *y*_*min*_, *y*_*int*_, *y*_*int1*_, *y*_*int2*_: specific *y* locations where the pore radius was determined
*m*, *m*_*i*_ (*i* = 1, 2, 3): gradient of porosity distribution laws
S: biophysical stimulus regulating the differentiation process
ɣ: octahedral shear strain
ʋ: interstitial fluid flow
*ɛ*_*I*_, ɛ_*II*_, ɛ_*III*_: principal strains
a: empirical constant *a* = 3.75%
b: empirical constant *b* = 3μms^-1^
*n*_*resorb*_, *n*_*mature*_, *c*: boundaries of the mechano-regulation diagram
A_lower_: lower bound of the pore radius variability range
A_upper_: upper bound of the pore radius variability range
V_i_bone_: volume of the generic element where the formation of mature bone is predicted to take place
n_b_: number of elements where the formation of mature bone is predicted to take place
V_BONE_: total volume of the elements where the formation of mature bone is predicted to take place
BO_%_: percentage of scaffold volume that is occupied by bone
Ω: objective function to optimize
PVPD: Percent Variation of the Pore Dimension
*A*_*H*_, *A*_*L*_: highest and lowest value of *A* along the *y*-axis, respectively
BO_%_tri-linear_: percentage of volume occupied by bone predicted for the tri-linear porosity distribution law
BO_%_constant_: percentage of volume occupied by bone predicted for the constant porosity distribution law
iBO_%_: increment of *BO*_*%*_
